# Dimensional Accuracy and Short-Term Stability of Orthodontic Resin-Printed Models: A Closed Dental System Compared with Commercial Desktop Workflows

**DOI:** 10.3390/dj14040220

**Published:** 2026-04-09

**Authors:** Pilar España-Pamplona, Davide Gentile, Adrian Curto-Aguilera, Riccardo Aiuto, Milagros Adobes-Martin, Daniele Garcovich

**Affiliations:** 1 Department of Dentistry, Universidad Europea de Valencia, 46010 Valencia, Spain; pilar.espana@universidadeuropea.es (P.E.-P.); milagros.adobes@universidadeuropea.es (M.A.-M.); 2 Department of Chemical Science and Technologies, Materials for Sustainable Development—Dentistry, University of Rome Tor Vergata, 00133 Rome, Italy; davide.gentile.dav@students.uniroma2.eu; 3 Department of Surgery, Universidad de Salamanca, 37007 Salamanca, Spain; adrian_odonto@usal.es; 4 Department of Biomedical, Surgical, and Dental Science, University of Milan, 20122 Milan, Italy; riccardo.aiuto@unimi.it

**Keywords:** orthodontics, 3D printing, vat photopolymerization, resin printers, dimensional accuracy, trueness, RMS deviation, CloudCompare^®^, digital workflow, model stability

## Abstract

**Background/Objectives:** Resin 3D printing is widely used to fabricate orthodontic diagnostic models, but the practical performance of commercial desktop workflows compared to dental-certified workflows is still debated. This study compared the dimensional accuracy and 7-day stability of maxillary orthodontic models printed from the same master STL file using a dental-certified workflow versus two commercial desktop workflows. **Methods:** An ISO 20896-1:2019-based reference cast with four 6 mm calibration spheres was used to generate a master STL file. Fifteen models were printed (n = 5 per workflow) using Primeprint™ (dental-certified workflow) and two commercial desktop printers (Anycubic Photon Mono M5s; Phrozen Sonic Mighty 14K REVO). The models were digitized at baseline (T0, ≤48 h) and after 7 days (T7) using a laboratory scanner. Surface superimposition in CloudCompare^®^ calculated the RMS (root mean square) surface deviation and mean signed deviation, and two calibrated operators performed independent extractions. **Results:** The mean RMS deviations were <0.10 mm for all workflows at both time points. No between-workflow differences were detected at T0 (H = 2.000; *p* = 0.368) or T7 (H = 1.520; *p* = 0.468), no within-workflow T0–T7 changes were significant (all *p* > 0.05), and the inter-operator agreement was excellent (ICC 0.991–0.999). **Conclusions:** Under the tested workflows, dental-certified and commercial desktop resin printing produced orthodontic models with a comparable global surface accuracy and short-term dimensional stability.

## 1. Introduction

Digital dentistry has rapidly expanded across dental specialties through the adoption of computer-aided design and manufacturing (CAD/CAM), intraoral scanning, and additive manufacturing workflows [1,2]. Intraoral scanners enable the non-invasive capture of dental arches and the generation of three-dimensional surface files (STL), which can be used for diagnosis, treatment planning, and the fabrication of physical models via 3D printing [2]. In orthodontic patients, digital full-arch impressions have demonstrated an accuracy comparable to conventional workflows and are often preferred by patients due to issues related to comfort and reduced chair time [3].

Additive manufacturing has expanded rapidly within the context of digital dentistry and is no longer limited to orthodontic diagnostic models. The recent literature establishes that 3D printing is increasingly being applied across prosthodontics, implantology, maxillofacial surgery, periodontics, orthodontics, and endodontics, particularly through patient-specific workflows based on digital imaging and CAD design [4,5]. In prosthodontics, beyond model fabrication, additive manufacturing has also been explored for fixed prosthetic applications such as crowns and bridges, although the degree of clinical translation still varies depending on the material system and indication [4,6]. In parallel, current evidence highlights that the performance of printed dental devices depends not only on the printer itself but also on the overall workflow, including the printing technology used, build orientation, material characteristics, and post-processing conditions [5,7].

For the purposes of the present study, a dental-certified workflow refers to a manufacturer-validated dental system that combines a printer, a dedicated dental material, and a validated post-processing protocol within a closed workflow, whereas a commercial desktop workflow refers to a desktop resin printing device operated according to manufacturer-recommended settings outside such a closed manufacturer-validated dental system; this distinction is consistent with the previous literature contrasting budget or non-dental systems with dental-specific printers, which highlights the relevance of calibrated resin settings, manufacturer-linked materials, and workflow parameters for dimensional accuracy [5,8].

Among the additive manufacturing technologies used in dentistry, vat photopolymerization systems (including stereolithography-based workflows) are widely adopted for the production of dental models, surgical guides, and appliances [5].

The dimensional accuracy of printed models is multifactorial and depends not only on the printer hardware but also on the print settings (e.g., layer thickness, build orientation, and support strategy), resin characteristics, and post-processing protocols (washing and post-curing) [7,8,9]. Several comparative studies have demonstrated that commercial desktop resin printing workflows can achieve acceptable dimensional accuracy for full-arch diagnostic models under controlled conditions, although their performance varies based on the technology and workflow [7,8,10]. Despite the growing use of low-cost printers in clinics and teaching environments, direct comparisons that explicitly contrast a closed, manufacturer-validated dental printing system with commonly implemented commercial workflows—while also assessing short-term dimensional stability—remain limited.

A reliable assessment of 3D accuracy requires robust digital tools for registration and surface comparisons. Open-source software such as 3D Slicer version 5.9 and CloudCompare^®^ version 2.13.2, as well as other validated platforms, allows a standardized superimposition and deviation analysis of 3D models and has been widely used in dentomaxillofacial research [11,12,13,14].

Therefore, the aim of this in vitro study was to compare the dimensional accuracy and short-term dimensional stability (within 7 days) of maxillary dental models printed from the same reference STL file using a dental-certified resin printing workflow (Primeprint™) and two commercial desktop resin printers (Anycubic Photon Mono M5s and Phrozen Sonic Mighty 14K REVO), each operated according to the respective manufacturer-recommended workflow. The null hypothesis was that no differences would be found between the tested workflows at either time point.

## 2. Materials and Methods

The present study follows an in vitro technical validation design. An anonymized maxillary arch STL file previously acquired as part of routine orthodontic records was used as the digital source; no additional clinical procedures were performed for research purposes. This project was approved by the Research Committee of the School of Doctoral Studies and Research at Universidad Europea de Valencia (Valencia, Spain) and was conducted in accordance with the Declaration of Helsinki [15].

### 2.1. Reference Model and Master STL File

**Figure 1:**
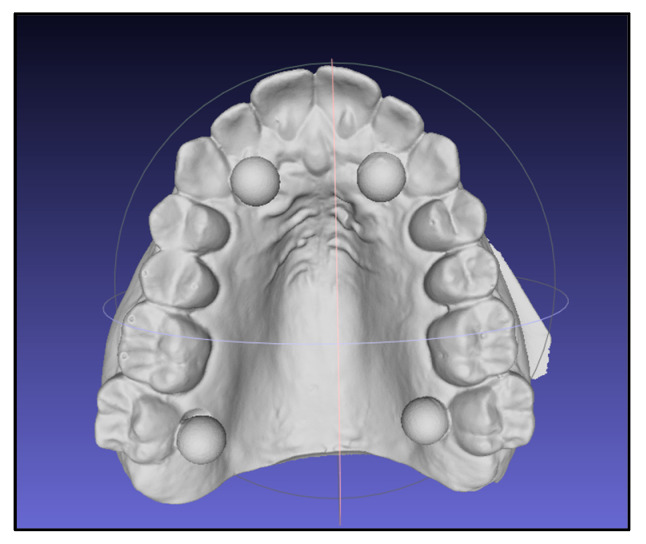
Reference cast and calibration spheres used to generate the master STL file based on the ISO 20896-1:2019 workflow.

A gypsum reference cast was fabricated and prepared following the principles described for accuracy assessment workflows in ISO 20896-1:2019, using external reference aids to support repeatable alignment and measurement [16]. Four calibrated spheres (6 mm diameter) were positioned on the cast to enable consistent registration and linear metrology in sagittal and transverse dimensions. The cast with spheres was digitized using an intraoral scanner (Primescan^®^, Dentsply Sirona, Bensheim, Germany). The resulting STL file was trimmed (Exocad^®^, exocad GmbH, Darmstadt, Germany) and used as the master file for all subsequent superimpositions and as the print file for all printers. A representative view of the master STL file and metrology spheres is shown in Figure 1.

### 2.2. 3D Printing Workflows

Three resin printing workflows were evaluated: one dental-certified workflow and two commercial desktop workflows. The dental-certified workflow used Primeprint™ (Dentsply Sirona, Bensheim, Germany) with the Primeprint Model resin (Dentsply Sirona, Bensheim, Germany). Printing and post-processing were performed following the manufacturer’s validated workflow, including automated washing, drying, and post-curing in the Primeprint Post-Processing Unit (PPU) with curing under a nitrogen atmosphere [17].

The commercial printers were Anycubic Photon Mono M5s (Anycubic, Shenzhen, Guangdong, China) and Phrozen Sonic Mighty 14K REVO (Phrozen Tech Co., Ltd., Hsinchu City, Taiwan), and both used ELEGOO Standard Photopolymer Resin V2.0 (Beige, 405 nm) (Shenzhen ELEGOO Technology Co., Ltd., Shenzhen, China), a standard non-dental photopolymer resin, and were operated following the resin and device manufacturers’ recommendations [18]. For the commercial workflow, models were oriented at 45° and generated with medium supports positioned to avoid palatal and buccal surfaces, minimizing the risk of surface alteration after support removal and finishing.

### 2.3. Post-Processing, Time Points, and Storage

For the commercial workflow, printed models were washed in isopropyl alcohol using the Anycubic Wash & Cure 3 Plus unit (Anycubic, Shenzhen, Guangdong, China) for 10 min, followed by 10 min of post-curing in the same unit after removing the wash container and placing the turntable base [19]. Primeprint™ models underwent the automated post-processing cycle within the closed PPU system described above [17].

To assess the short-term dimensional stability during the first week, each printed model was digitized at two time points: baseline (T0) and 7 days after printing (T7). The T0 scan was performed within 48 h after printing and post-processing for all groups to minimize uncontrolled early volumetric changes prior to digitization. Between T0 and T7, models were stored at room temperature and protected from direct light.

### 2.4. Sample Size Calculation and Digitization

An a priori precision-based sample size calculation was performed using the variability reported by Nulty et al. for mean dimensional deviation outcomes in resin printing (typical SD ≈ 0.04 mm) [8]. This study was designed to estimate the mean deviation per workflow with a 95% confidence interval half-width of approximately 0.05 mm (50 μm), which is considered adequate to clinically assess relevant differences in orthodontic diagnostic models. Assuming an SD ≈ 0.04 mm, five prints per printer yield an expected 95% CI half-width of ~0.05 mm (t{0.975,4} × SD/√n ≈ 2.776 × 0.04/√5 ≈ 0.050 mm), confirming the reliable estimation of the mean deviation within the tested workflows.

Five prints were produced per printer (n = 5), resulting in 15 printed models. Each model was digitized twice (T0 and T7), yielding 30 STLs for analysis. All scans were performed with a laboratory scanner (E2, 3Shape, Copenhagen, Denmark) according to the manufacturer’s instructions and calibration procedures [20] by the same operator using a standardized protocol.

### 2.5. Surface Registration and Deviation Analysis

All printed model STLs were imported into CloudCompare^®^ (v2.11; open source project) and superimposed onto the master STL file. The initial alignment was performed using the calibration spheres as reference landmarks, followed by iterative closest point (ICP) refinement. Deviation analyses were computed as point-to-surface distances and summarized using the root mean square (RMS), surface deviation (mm), and mean signed deviation (mm). The RMS was defined as the square root of the mean of the squared point-to-surface distances and was considered the primary global accuracy metric (lower values indicate closer overall agreement with the master STL file). Color maps and corresponding histograms were generated for each superimposition. To reduce the influence of outliers, a deviation display range of ±0.30 mm was applied; points outside this range were treated as outliers for visualization/noise control, consistent with protocols used in previous 3D dental accuracy studies [11,12,13,14].

### 2.6. Measurement Reliability and Data Handling

Two calibrated operators independently performed the CloudCompare^®^ workflow and extracted deviation metrics from all superimpositions. Prior to this study, both operators completed calibration training using at least 100 trial measurements each. The inter-operator agreement was assessed using the intraclass correlation coefficient (ICC), and a maximum acceptable inter-operator deviation of 0.05 mm was predefined for extracted metrics.

For inferential analyses, the printed model (print) was the unit of analysis (n = 5 per workflow). To avoid pseudo-replication, operator values were averaged per print at each time point (T0 and T7) prior to statistical testing.

### 2.7. Statistical Analysis

Statistical analyses were performed using SPSS Statistics (version 25.0; IBM Corp., Armonk, NY, USA) with α = 0.05. Data normality was evaluated via the Shapiro–Wilk test. When normality assumptions were not met, between-workflow comparisons were performed using the Kruskal–Wallis test, and paired time point comparisons within each workflow were performed using the Wilcoxon signed-rank test.

Inter-operator agreement was quantified via the ICC.

## 3. Results

**Figure 2:**
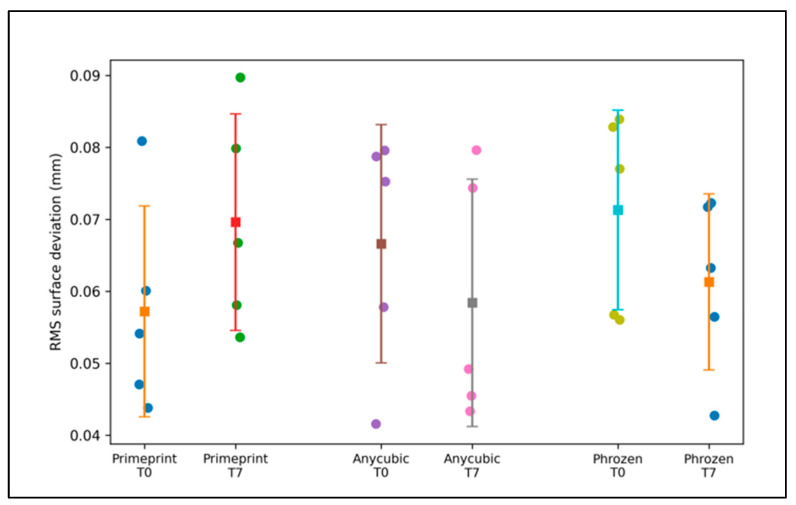
RMS deviation relative to the master STL file by workflow and time point. Individual dots represent the five printed models per group (n = 5); colors are used only to visually distinguish individual prints and do not encode additional variables. Squares indicate the mean and error bars represent the 95% confidence interval.

The dimensional accuracy outcomes relative to the master STL file are summarized in Table 1 and illustrated in Figure 2. Overall, the RMS surface deviation values were low for all three workflows and remained consistently below 0.10 mm at both time points, indicating small global surface discrepancies relative to the reference model (Table 1). At baseline (T0), the dental-certified workflow (Primeprint™) exhibited a mean RMS deviation of 0.0572 ± 0.0147 mm, whereas the commercial workflows presented slightly higher mean RMS values of 0.0666 ± 0.0165 mm (Anycubic) and 0.0713 ± 0.0139 mm (Phrozen). After 7 days (T7), the mean RMS deviation was 0.0696 ± 0.0150 mm for Primeprint™, while the commercial workflows had mean RMS values of 0.0584 ± 0.0172 mm (Anycubic) and 0.0613 ± 0.0122 mm (Phrozen) (Table 1). Although the rank order of mean RMS values differed between T0 and T7, the magnitude of these differences was small, and the variability within each workflow was comparable.

These findings are reflected in Figure 2, where the distributions at both time points largely overlap, and no clear separation between the dental-certified and commercial workflows is visually apparent. In addition to the RMS, the mean signed deviation values were small (≤0.063 mm) and consistently positive across workflows, suggesting only a minor directional bias relative to the master STL file (Table 1), suggesting the absence of a consistent systematic bias toward model expansion or contraction relative to the master STL file.

The between-workflow comparisons are presented in Table 2. Consistent with the overlapping distributions observed in Figure 2, the Kruskal–Wallis tests revealed no statistically significant differences in the RMS deviation among the three workflows at either time point (T0: H = 2.000, *p* = 0.368; T7: H = 1.520, *p* = 0.468) (Table 2). In combination with the narrow absolute differences observed in Table 1, these results indicate that, under the tested conditions, the dental-certified workflow and the two commercial desktop workflows achieved a comparable global surface agreement relative to the master STL file at both the baseline and day 7.

**Figure 3:**
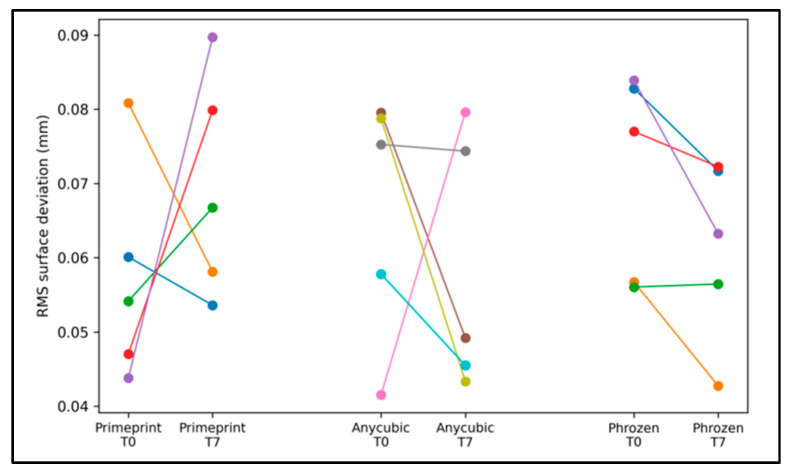
Paired RMS deviation at T0 and T7 for each printed model within each workflow.

The short-term dimensional stability during the first week is summarized in Table 3 and visualized in Figure 3. Within-workflow changes were expressed as paired differences per print (T7–T0). The direction of change was small and not uniform across groups: Primeprint™ demonstrated a modest tendency toward a higher RMS at T7, whereas the commercial workflows exhibited slight reductions in the RMS at T7 compared with T0 (Table 3). However, these changes were minor in magnitude, and none reached statistical significance (all *p* > 0.05), indicating that there was no meaningful short-term distortion over the first week within any workflow. The paired trajectories shown in Figure 3 further support this interpretation, with the within-workflow variability across prints exceeding any systematic time effect.

The measurement reproducibility is reported in Table 4. The inter-operator agreement was excellent across workflows (ICC range: 0.991–0.999), supporting the robustness of the deviation metric extraction. The mean absolute inter-operator difference for the RMS extraction was 0.0010 mm, and the maximum observed difference was 0.0043 mm, indicating that the operator-related variability was negligible compared with the observed between-print variability and did not materially influence the workflow comparisons.

## 4. Discussion

This study evaluated the dimensional accuracy of 3D-printed orthodontic models produced via three resin printing workflows from different market segments (one dental-certified workflow and two commercial desktop workflows). All printed models were compared to a master STL file using surface superimposition and deviation analysis in CloudCompare^®^. Deviation metrics (including mean deviation, standard deviation, and root mean square [RMS]) were extracted by two calibrated operators. Overall, the results revealed no statistically significant differences between workflows in the main deviation outcomes at either time point, indicating that all three workflows produced models with a comparable dimensional accuracy within a range that is commonly considered clinically acceptable for orthodontic diagnostic models.

No relevant differences were identified between the two operators, as evidenced by excellent inter-operator reliability (ICC >0.991 across workflows). This confirms the high reproducibility of the adopted procedure and supports the use of calibration spheres to facilitate repeatable registration—an approach that has been reported to improve consistency in similar accuracy studies and is consistent with ISO-based accuracy assessment principles [16]. From a translational perspective, this is particularly relevant because clinical and laboratory digital workflows often involve multiple technicians; a highly reproducible analysis approach reduces the risk that observed differences are due to measurement variability rather than true manufacturing performance.

The use of CloudCompare^®^ enabled robust surface-based comparisons, and similar approaches have been adopted in studies evaluating multiple printers under standardized digital protocols [8]. Those investigations revealed that some low-cost printers can achieve results statistically comparable to more expensive devices for at least some outcome dimensions, supporting the notion that recent improvements in desktop resin printing may narrow the performance gap between market segments. Our findings extend this concept to a controlled orthodontic diagnostic model workflow, where no relevant differences were observed when a rigorous registration and analysis protocol was applied.

The accuracy and precision values observed remained within the limits commonly cited as clinically acceptable (often around <100 μm, depending on indication), which is consistent with previous research that compared different printing approaches, including CLIP and DLP systems [21]. Although no statistically significant differences were detected in our comparisons, descriptively, the dental-certified workflow tended to show slightly lower mean deviation and RMS values than one of the desktop workflows. This trend is in line with reports stating that professional-grade systems may perform marginally better than economical devices while still maintaining all outputs within clinically acceptable ranges [22]. However, it is important to emphasize that, in practice, the final accuracy reflects the entire workflow (printer, resin, parameters, and post-processing), and small descriptive differences may not translate into clinically meaningful discrepancies.

Nevertheless, even when the overall agreement appears to be acceptable, we cannot rule out the possibility that variables such as the print orientation, support strategy, exposure parameters, resin composition, and post-curing conditions influence outcomes and may affect specific clinical use cases. Prior work suggests that technologies such as PolyJet and DLP may provide higher dimensional accuracy than other additive approaches in certain settings, although outputs across modalities can still be clinically valid depending on the application [23]. In addition, comparative studies focusing on DLP vs. LCD indicate that both technologies may achieve acceptable accuracy, but differences can depend on workflow parameters and post-processing conditions rather than the “technology” alone [24,25]. This supports our decision to interpret these findings as comparisons of tested workflows, rather than as general statements about printer generations or resin printing technology as a whole.

Regarding temporal behavior, time-dependent dimensional changes have been reported at longer intervals after printing and may be influenced by storage conditions [26]. In the present study, models were rescanned within 48 h and again at 7 days, and no significant within-workflow differences were observed. Therefore, while our findings confirm short-term stability under the studied conditions, future studies should extend the follow-up period and standardize or test storage environments more explicitly [27].

This study has several strengths: the use of a structured surface-based analysis approach, the application of non-parametric tests when normality assumptions were not met, and excellent inter-operator agreement. Additionally, the inclusion of a short-term stability assessment via rescans of the same models provides clinically relevant information for workflows that use printed models within days. More broadly, evidence syntheses, such as network meta-analyses, highlight that accuracy is driven by multiple interacting workflow factors and that several resin printing approaches may achieve acceptable performances depending on parameterization [27]. Systematic reviews in orthodontics similarly support the expanding clinical applicability of 3D printing while emphasizing the importance of workflow control and indication-specific accuracy requirements [24,28].

As for limitations, the sample size was limited to five prints per workflow, and the prints were derived from a single maxillary STL file, which limits the generalizability across different anatomies and degrees of complexity. In addition, the commercial desktop workflows used ELEGOO Standard Photopolymer Resin V2.0, a standard non-dental resin, whereas the Primeprint™ workflow used a manufacturer-validated dental model resin within a closed dental system. This represents a key limitation of the present study, as the resin formulation and post-processing conditions may influence dimensional accuracy independently of the printer hardware. Accordingly, the present comparison should be interpreted at the workflow level rather than as evidence of the equivalence between printers alone. Future research should explore longer-term dimensional stability, different storage environments, multiple anatomies, and alternative materials and parameter sets to clarify which workflow components most strongly influence clinically relevant deviation patterns.

## 5. Conclusions

Considering the limitations of this in vitro validation study, the tested resin printing workflows—one dental-certified workflow and two commercial desktop workflows using different materials and post-processing protocols—produced diagnostic orthodontic models with a comparable dimensional accuracy under the experimental conditions, with mean RMS surface deviations below 0.10 mm and no significant differences between workflows at the baseline or after 7 days.

The inter-operator agreement in this study was excellent (ICC > 0.99), supporting the reproducibility of the registration and deviation-analysis protocol. Short-term dimensional stability under this study’s storage conditions was confirmed, although a longer follow-up and multiple anatomies are needed before generalizing these findings to other clinical indications or extended storage periods.

## Figures and Tables

**Table 1:** RMS deviation and mean signed deviation relative to the master STL file by workflow and time point (operator-averaged; n = 5 prints per workflow).

Workflow	TP	n	RMS (Mean ± SD)	95% CI	Mean Dev.
Primeprint™	T0	5	0.0572 ± 0.0147	0.0390 to 0.0754	0.0494
Primeprint™	T7	5	0.0696 ± 0.0150	0.0509 to 0.0883	0.0627
Anycubic	T0	5	0.0666 ± 0.0165	0.0460 to 0.0871	0.0476
Anycubic	T7	5	0.0584 ± 0.0172	0.0371 to 0.0797	0.0517
Phrozen	T0	5	0.0713 ± 0.0139	0.0541 to 0.0885	0.0514
Phrozen	T7	5	0.0613 ± 0.0122	0.0461 to 0.0765	0.0486

TP = time point (T0 = baseline; T7 = 7 days). RMS = root mean square surface deviation. Mean Dev. = mean signed deviation. Values are expressed in millimeters (mm). Primeprint™ = dental-certified workflow. Anycubic and Phrozen = commercial workflows.

**Table 2:** Between-workflow comparisons of RMS deviation at each time point.

TP	H	df	*p*
T0	2.000	2	0.368
T7	1.520	2	0.468

TP = time point (T0 = baseline; T7 = 7 days). Between-workflow comparisons were performed using the Kruskal–Wallis test. Significance level α = 0.05.

**Table 3:** Within-workflow comparisons between T0 and T7 for RMS deviation (operator-averaged; paired by print; n = 5 prints per workflow).

Workflow	Median Δ (T7–T0)	Q1–Q3	W	*p*
Primeprint™	0.0126	−0.0065 to 0.0328	4.0	0.438
Anycubic	−0.0123	−0.0304 to −0.0009	5.0	0.625
Phrozen	−0.0111	−0.0140 to −0.0047	1.0	0.125

Δ = paired difference in RMS deviation (T7 − T0). Q1–Q3 = first to third quartile. Within-workflow comparisons were performed using the Wilcoxon signed-rank test (paired by print). Values are expressed in millimeters (mm). Primeprint™ = dental-certified workflow; Anycubic and Phrozen = commercial workflows. Significance level α = 0.05.

**Table 4:** Inter-operator reliability for deviation metric extraction (intraclass correlation coefficient, ICC).

Workflow	ICC	95% CI	*p*-Value
Primeprint™	0.999	0.993 to 1.000	<0.001
Anycubic	0.999	0.990 to 1.000	<0.001
Phrozen	0.991	0.915 to 0.999	<0.001

ICC = intraclass correlation coefficient (two-way random effects model, absolute agreement). 95% CI = 95% confidence interval. ICC was calculated to assess inter-operator agreement for deviation metric extraction. Primeprint™ = dental-certified workflow; Anycubic and Phrozen = commercial workflows. Significance level α = 0.05.

## Data Availability

The data supporting the findings of this study are available from the corresponding author upon reasonable request.
